# Acute and Chronic Responses of Aerobic Exercise With Blood Flow Restriction: A Systematic Review

**DOI:** 10.3389/fphys.2019.01239

**Published:** 2019-10-04

**Authors:** Júlio Cesar Gomes Silva, Elísio Alves Pereira Neto, Patrick Alan Souza Pfeiffer, Gabriel Rodrigues Neto, Amanda Santos Rodrigues, Michael G. Bemben, Stephen D. Patterson, Gilmário Ricarte Batista, Maria S. Cirilo-Sousa

**Affiliations:** ^1^Associate Graduate Program in Physical Education, Department of Physical Education, Federal University of Paraíba, João Pessoa, Brazil; ^2^Laboratory of Kinanthropometry and Human Performance, Department of Physical Education, Federal University of Paraíba, João Pessoa, Brazil; ^3^Faculty Nova Esperança (FAMENE/FACENE), Coordination of Physical Education, Nursing and Medical Schools, João Pessoa, Brazil; ^4^Coordination of Physical Education, University Center for Higher Education and Development (CESED/UNIFACISA/FCM/ESAC), Campina Grande, Brazil; ^5^Alliance for Research in Exercise, Nutrition and Activity (ARENA), School of Health Sciences, University of South Australia, Adelaide, SA, Australia; ^6^Department of Health and Exercise Science, Norman, OK, United States; ^7^Faculty of Sport, Health and Applied Science, St Marys' University, London, United Kingdom

**Keywords:** aerobic exercise, blood flow restriction, elderly, adults, hemodynamic

## Abstract

This study systematically reviewed the available scientific evidence pertaining to the acute and chronic changes promoted by aerobic exercise (AE) combined with blood flow restriction (BFR) on neuromuscular, metabolic and hemodynamic variables. PubMed, Web of Science^TM^ and Scopus databases were searched for the period from January 2000 to June 2019 and the analysis involved a critical content review. A total of 313 articles were identified, of which 271 were excluded and 35 satisfied the inclusion criteria. Twelve studies evaluated the acute effects and eight studies evaluated the chronic metabolic effects of AE + BFR. For the neuromuscular variables, three studies analyzed the acute effects of AE + BFR and nine studies analyzed the chronic effects. Only 15 studies were identified that evaluated the hemodynamic acute effects of AE + BFR. The analysis provided evidence that AE combined with BFR promotes positive acute and chronic changes in neuromuscular and metabolic variables, a greater elevation in hemodynamic variables than exercise alone, and a higher energy demand during and after exercise. Since these alterations were all well-tolerated, this method can be considered to be safe and feasible for populations of athletes, healthy young, obese, and elderly individuals.

## Introduction

The American College of Sports Medicine recommends adults to routinely perform moderate-intensity aerobic exercise 5–7 days a week (40–60% of VO_2_ peak) or vigorous exercise 3 days a week (≥60% VO_2_ peak) in order to improve cardiorespiratory fitness and reduce the risk of metabolic, cardiovascular and pulmonary diseases (Garber et al., 2011). Aerobic exercise (AE) is considered a successful method for improving physical fitness in healthy individuals (Clemente Suárez and González-Ravé, 2014) as well as in individuals with special limitations (Kelley and Kelley, 2008).

However, high-intensity AE, requires intense exertion and involves high mechanical stress, which makes it difficult to perform this exercise modality for elderly, those recovering from orthopedic injuries or individuals afflicted with chronic illness. Thus, a possible alternative to high-intensity aerobic training is a method that combines AE (walking or cycling) with blood flow restriction (BFR). BFR exercise involves the use of inflatable cuffs or elastic bands positioned at the proximal region of the exercising musculature (upper arms or upper legs) and is performed at low exercise intensities ranging between 20 and 40% of maximum oxygen consumption (VO_2max_) and for short durations. AE combined with BFR (AE + BFR) promotes significant changes in neuromotor and cardiorespiratory activities that are similar to those often observed with high intensity exercise (Abe et al., 2006, 2010a; Pope et al., 2013).

AE + BFR has been shown to elicit neuromuscular (Abe et al., 2006; Ozaki et al., 2010; Corvino et al., 2014; Kim et al., 2016), metabolic (Abe et al., 2010b; Ozaki et al., 2010; Park et al., 2010; Loenneke et al., 2011; Kim et al., 2016; Taylor et al., 2016; Silva et al., 2019) and cardiovascular (Abe et al., 2010a; Ozaki et al., 2010; Renzi et al., 2010; Kumagai et al., 2012; Karabulut and Garcia, 2015; Silva et al., 2018) adaptations in healthy adults, obese, elderly individuals, and athletes. However, a consensus on the protocols that are used to perform AE + BFR has not been attained. A consensus would establish the actual effectiveness of this type of intervention on the acute responses and chronic adaptations of AE + BFR on metabolic, neuromuscular and hemodynamic variables. Therefore, the objective of this review was to systematically analyse the available scientific evidence regarding the acute responses and chronic adaptations promoted by AE + BFR on metabolic, neuromuscular, hemodynamic variables in athletes, healthy adults, and obese, elderly individuals.

## Methods

To conduct the searches, the following English descriptors were employed: (“aerobic exercise” OR “cycling” OR “walking”) AND (“kaatsu” OR “vascular occlusion” OR “blood flow restriction” OR “kaatsu training”). The following inclusion criteria were adopted: original research that was conducted human subjects, research published in journals indexed in the selected databases, and research that contained individuals in the age range from 18 to 80 years and evaluated the acute and chronic changes promoted by AE + BFR. Articles were excluded if they (a) had scores lower than six on the Physiotherapy Evidence Database (PEDro) scale; (b) review articles; (c) articles that used protocols with strength exercises; (d) articles of viewpoints/opinions, studies validation; (e) book chapters, theses or dissertations and (f) case study articles.

Two investigators (JC and EP) conducted the on-line search in an independent and blind manner; their findings were subsequently compared. In the case of disagreement, a third evaluator (GR) established a consensus. During screening, the title and abstract of the identified articles were read. Thus, studies in which the title and abstract provided sufficient information were obtained. All articles were then read in their entirety. The references of these articles were reviewed to identify other potentially relevant studies that had not been identified in the electronic search.

### Methodological Quality: PEDro Scale

Currently, the most used scale in the area of rehabilitation is the PEDro scale (http://www.pedro.fhs.usyd.edu.au), which was developed by the Physiotherapy Evidence Database for use with regard to experimental studies. This scale has a maximum total score of 10 points and includes criteria concerning the evaluation of internal validity and the presentation of the statistical analysis used. For each criterion defined in the scale, one point (1) are attributed to the presence of indicators regarding the quality of the evidence presented, and zero points (0–0) are attributed to its absence. The PEDro scale is composed of the following elements: (a) the specification of inclusion criteria (item not scored); (b) random allocation; (c) concealment of allocation; (d) similarity of the groups at baseline or the initial phase; (e) blinding of all participants; (f) blinding of all therapists; (g) blinding of all assessors; (h) measures of at least one primary outcome obtained from more than 85% of participants allocated; (i) “intention to treat” analysis; (j) between-group comparisons of at least one primary outcome; and (k) reports of measures of variability and estimations of parameters concerning at least one primary variable (Maher et al., 2003).

This study was conducted according to the Preferred Reporting Items for Systematic Reviews and Meta-Analyses (PRISMA) guidelines (Liberati et al., 2009). Data analysis was performed based on a critical review of the content using the following criteria: title, abstract, rationale, objectives, protocol, risk of bias across studies, study characteristics, individual study results, limitations, and conclusions.

## Results

**Figure 1:**
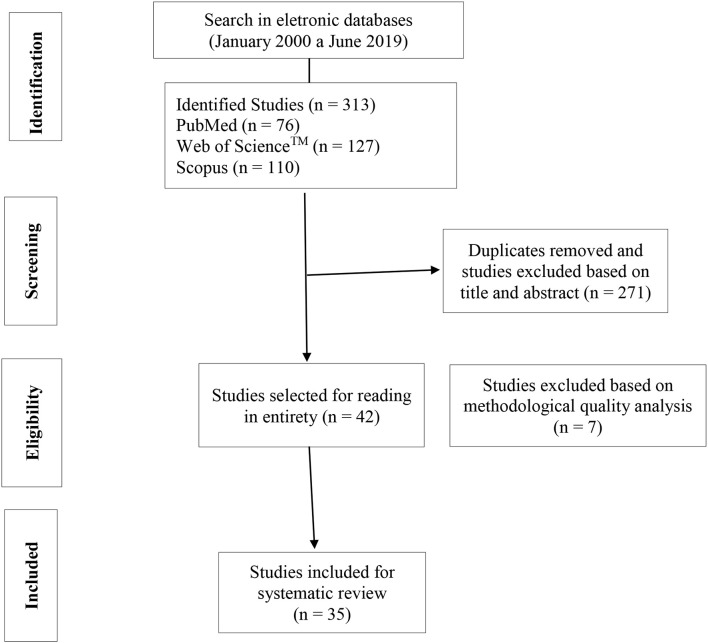
Flowchart of the study selection process.

The synthesis of the studies' results was presented based on a structured script that considered the following components: (a) author (year) of the study; (b) subjects; (c) variable; (d) exercise protocol; (e) intensity; (f) exercise volume; g) interval between sets; (h) BFR pressure used during exercise; (i) BFR time, and (j) cuff width and k) main results. Of the 313 articles that were identified, 271 (86.2%) articles were excluded based on their title and abstract. Thus, 42 articles were selected to be read in their entirety. Thirty five articles were selected after applying the eligibility criteria. This process was based on the analysis of the methodological quality of the studies. A systematic evaluation of the changes promoted by AE combined with BFR in metabolic, neuromuscular and hemodynamic variables was performed (Figure 1).

## Effect of AE + BFR on Metabolic Variables

Based on the analysis of the studies that investigated AE + BFR, eleven studies evaluated the acute effect of this training method on metabolic variables (Ozaki et al., 2010; Loenneke et al., 2011, 2012; Mendonca et al., 2014, 2015; Karabulut and Garcia, 2015; Corvino et al., 2016; Conceição et al., 2018; Thomas et al., 2018; Pfeiffer et al., 2019; Silva et al., 2019) and nine studies evaluated the chronic effect of this training method on metabolic variables (Abe et al., 2010a,b; Park et al., 2010; Corvino et al., 2014; de Oliveira et al., 2015; Kim et al., 2016; Taylor et al., 2016; Paton et al., 2017; Tanaka and Takarada, 2018).

Studies that investigated the acute effect of AE + BFR assessed oxygen consumption (VO_2_) during exercise (Ozaki et al., 2010; Loenneke et al., 2011; Corvino et al., 2016; Thomas et al., 2018; Silva et al., 2019), energy expenditure during exercise (Loenneke et al., 2011; Mendonca et al., 2014; Karabulut and Garcia, 2015; Conceição et al., 2018; Pfeiffer et al., 2019) blood lactate concentration during exercise (Loenneke et al., 2012; Corvino et al., 2016; Thomas et al., 2018) and only one study investigated (Mendonca et al., 2015) excessive post-exercise oxygen consumption (EPOC). Regarding the chronic effects of AE + BFR, some studies evaluated cardiorespiratory capacity (Abe et al., 2010a,b; Park et al., 2010; de Oliveira et al., 2015; Kim et al., 2016; Taylor et al., 2016; Tanaka and Takarada, 2018), time to exhaustion in severe exercise (Corvino et al., 2014; Paton et al., 2017), maximum power and onset blood lactate accumulation (de Oliveira et al., 2015).

## Acute Effects of AE + BFR

### Oxygen Consumption *(l/min)*

Ozaki et al. (2010), conducted a study using a crossover model: ten young men performed two randomized cycling protocols on a cycle ergometer that was composed of four stages. Each protocol was performed with duration of 4 min, a constant speed of 70 revolutions per minute (rpm) and progressive intensities of 0, 20, 40, and 60% of VO_2max_. The results indicated that VO_2_ during cycling with BFR at an intensity of 20% of VO_2max_ exceeded the oxygen consumption for the cycling protocol without the use of BFR. Mendonca et al. (2014), found that exercise performed with BFR had a higher energy cost than the session performed at the same intensity without BFR. Similar results were observed by Silva et al. (2019), which indicated that the VO_2_ during walking with BFR at an intensity of 40% of VO_2peak_ exceeded the oxygen consumption for the walking protocol without BFR.

### Energy Expenditure

Regarding the impact of BFR on energy expenditure (EE), two studies revealed that the performance of interval walking combined with BFR has a higher energy demand than walking without BFR. In both studies, energy expenditure (EE) was calculated using the caloric equivalents for the non-protein respiratory exchange ratio (RER) values for each liter of oxygen used. The unit of measure used was the kilocalorie (Kcal). Loenneke et al. (2011) found that exercise performed with BFR yielded higher VO_2_ elevation and energy expenditure than the session with the same protocol without the use of elastic bands. A study by Karabulut and Garcia (2015) was performed using a crossover model: 34 obese people 18 men and 16 women performed three randomized cycling sessions with a speed of 50 rpm and a constant load of 50 Watts in two sets of 10 min and a 1-min interval between sets. An exercise protocol was performed without BFR, and two protocols were performed with BFR and inflatable cuffs in the thigh; demonstrating that exercise with BFR significantly increased energy demand during exercise.

### Excessive Post-exercise Oxygen Consumption (EPOC)

Only one study investigated the impact of AE + BFR on excessive post-exercise oxygen consumption (Mendonca et al., 2015). These authors observed that the exercise performed with BFR significantly increased the magnitude of EPOC compared to a session with the same intensity without the use of BFR.

### Blood Lactate Concentration

In a study by Loenneke et al. (2012), the volunteers performed two random sessions of interval walking on a treadmill with five sets of 2 min and a 1-min interval between sets, with a constant speed of 75 meters per minute (m/min). The stimulus generated by walking with BFR was not sufficient for increasing post-exercise metabolic stress. However, separate studies by Corvino et al. (2016) and Thomas et al. (2018), both observed that AE + BFR increased the concentration blood lactate more than a AE session without BFR.

## Chronic Effects of AE + BFR

### Cardiorespiratory Capacity

Abe et al. (2010b) conducted a study with 19 active elderly individuals, including four men and 15 women (60–78 years old) who were randomly divided into two groups: walking with BFR (W + BFR; *n* = 11) and walking without BFR (CONTROL; *n* = 8). Both groups performed 20 min of continuous walking 5 days per week for 6 weeks. W + BFR did not promote improvement in the cardiorespiratory capacity. In the study by Park et al. (2010), 12 college basketball players were randomly divided into two groups: (1) interval walking with BFR (IW + BFR; *n* = 7), and (2) interval walking without BFR (CONTROL, *n* = 5). Both groups performed five sets of 3 min with a 1-min interval between sets, speeds between 4 and 6 km/h and a 5% incline on the treadmill two times a day, six times a week for 2 weeks. A significant increase in the cardiorespiratory capacity of the basketball players was observed for the walking with BFR group.

The cycling protocol combined with BFR in the study by Abe et al. (2010a) promoted an increase in the cardiorespiratory capacity of young men after 8 weeks. Similarly, improvements in cardiorespiratory capacity of 20 trained men after 4 weeks of interval sprints with BFR were observed (Taylor et al., 2016). However, that was not observed in 31 trained college students who performed 20 min of cycling with BFR at 30% of HR_max_ three times a week for 6 weeks (Kim et al., 2016) (Table 1).

**Table 1:** Synthesis of the results of studies that evaluate the acute and chronic effects of combined aerobic exercise and blood flow restriction on metabolic variables in obese individuals, athletes and young adults.

**References**	**Subjects**	**Variables**	**Exercise protocol**			**Intensity or frequency of training/duration of study**	**Exercise volume**		**Interval**	**PADE** **(mmHg)**	**BFR between sets**	**SW** **(cm)**	**Results**
			**EX**	**FE**	**SE**		**Sets or continuous**	**Repetitions or duration time**					
Abe et al., 2010b	19 Elderly men and women	VO2_max_	WALK	TREADMILL	67 m/min	5× week/6 weeks	Continuous	20 min	-	200	–	5.0	No improvement in VO_2Max_ in the groups
Abe et al., 2010a	19 Adult men	VO2_max_	CYC	CYCLE	–	40% VO_2_ with and without BFR 1× day/3× week - 8 weeks	Continuous	15 min with BFR 45 min without BFR	–	160–210	–	5.0	↑ VO_2Max_ in cycling with BFR
Park et al., 2010	12 Basketball players	VO2_max_ VE_Max_	WALK	TREADMILL	4–6 km/h	2× day/6× week 2 weeks	5 Series	3 min	1 min	160–230	Yes	–	↑VE_max_ and VO_2max_ in training protocol with BFR
Ozaki et al., 2011	10 Men	VO_2_ (L/min)	CYC	CYCLE	70 rpm	20–40–60% VO_2max_ with and without BFR	Continuous	4 min each stage	–	200	Yes	5.0	↑VO_2_ between 20 and 60% in the session with BFR
Loenneke et al., 2011	10 Men and women	EE and VO_2_ (L/min)	WALK	TREADMILL	75 m/min	AE + BFR AE	5 sets	2 min	1 min	Elastic	Yes	7.6	↑VO_2_ and EE in the exercise session with BFR
Loenneke et al., 2012	9 Men and women	[La^+^]	WALK	TREADMILL	75 m/min	AE + BFR AE	5 sets	2 min	1 min	Elastic	Yes	7.6	WALKING+BFR does not change metabolic stress
Mendonca et al., 2014	18 Healthy men	Energy expenditure	WALK	TREADMILL	–	<30%VO_2max_	5 sets	3 min	1 min	200	Yes	6 × 83	Energy expenditure > for walking with BFR
Corvino et al., 2014	13 Active subjects	TTE	CYC	CYCLE	–	30% P_max_ + BFR 30% P_max_	2 Set	5–8 Repetitions	1 min	140–200	Yes	18	↑ in TTE for walking with BFR
Mendonca et al., 2015	17 Young men	EPOC	WALK	TREADMILL	–	<30%VO_2Max_	5 Sets	3 min	1 min	200	Yes	6 × 83	EPOC> in exercise session with BFR
de Oliveira et al., 2015	37 Subjects	Pmax. PALS, VO_2Max_	CYC	CYCLE	–	30% P_max_ + BFR110% P_max_30% P_max_110%-30% P_max_ +BFR3x week/4 weeks	2 Sets	5–8 Repetitions	5 min	140–200	Yes	18	↑VO_2Max_ and P_max_ and improvement in PALS for the LI with BFR protocol
Karabulut and Garcia, 2015	34 Obese men and women	EE, RER	CYC	CYCLE	50 rpm	40%+BFR 60%+BFR Without BFR 50 Watts	2 Sets	10 min	1 min	40% 60%	Yes	5.0	↑ EE and RER > in the BFR protocol
Taylor et al., 2016	20 Trained men	VO_2Max_	RUN	TREADMILL	–	SIT + BFR SIT 2× week/4 weeks	4, 5, 6. and 7 sprints	30 s	270 s	130 post-exercise	No	6 × 83	↑VO_2Max_ for the protocol with BFR
Kim et al., 2016	31 Trained young men	VO_2Max_	CYC	BICYCLE	–	3× week/6 weeks	Continuous	20 min	–	160–180	Yes	5.0	VO_2__MAX_ did not increase in the LI group.
Corvino et al., 2016	12 Men	VO_2_, [La], VE	CYC	CYCLE	–	HI IBFR30 CBFR30 CON30 IBFR0	2 Sets with 5 reps Continuous	2 35 min	1	80%	No	18	↑ metabolic strain and muscle deoxigenation in protocol I-BFR30
Paton et al., 2017	10 men 6 women	RE, TTE and PRV	RUN	TREADMILL	80%PRV	AE + BFRCON 2× week/4 weeks	2 Sets	5 repetitions	30 sec	Elastic	Yes	7–10	↑ RE, TTE and PRV in protocol with BFR
Conceição et al., 2018	12 Men	TEE, ALM, AM, VE	CYC	CYCLE	60–70 rpm	40% VO_2Max_ + BFR 40% VO_2Max_	Continuous	30 min	–	80%	No	18	↑ metabolic anaerobic, aerobic, total energy expenditure and Ventilation in protocol AE + BFR
Thomas et al., 2018	18 Men	VO_2_ and [La]	CYC	CYCLE	–	HI 85%Pmax LI 40% LI + BFR	3 Sets	2 min	2 min active	80%	Yes	10	↑VO_2_, [La] in protocol HI > LI+BFR and LI. VO_2_, [La] in protocol LI+BFR > LI
Tanaka and Takarada, 2018	30 Elderly men	VO_2Max_	CYC	CYCLE	–	40–70%VO_2Max_ + BFR 3× week/6 months	Continuous	15 min	–	40–80 mmhg ↑ SBP	No	9 × 7	↑ VO_2Max_ in protocol AE + BFR
Silva et al., 2019	22 Men	VO_2_	RUN WALK	TREADMILL	–	HIIE AE + BFR AE	6 Sets Continuous	90 s 18 min	90 s active	50%	No	18	↑VO_2_ in protocol HIIE But, VO_2_ in protocol AE + BFR > AE
Pfeiffer et al., 2019	24 Men	EE	WALK	TREADMILL	–	40% Maximal Speed	5 Sets	2 min	1 min	0 50% 80% 100%	Yes	18	↑EE in protocol walk with 50% BFR

*BFR, blood flow restriction; VO_2Max_, maximum oxygen consumption; VE_MAX_, Maximum ventilation; P_MAX_, maximum power; EPOC, excessive post-exercise oxygen consumption; EE, energy expenditure; TEE, total energy expenditure; ALM, Anaerobic Lactic Metabolism; RE, running economy; TTE, time to exhaustion; AM, Aerobic Metabolism; VE, Ventilation; PRV, peak running velocity; RER, respiratory exchange ratio; OBLA, onset blood lactate accumulation; EX, exercise; FE, form of execution; SE, speed of execution; WALK, walking; CYC, cycling; RUN, running; CYCLE, cycle ergometer; min, minutes; s, seconds; CON, Control; PADE, pressure applied during the exercise; SW, sleeve width; –, Not informed; ↑, significant increase; LI + BFR, low intensity combined with blood flow restriction; LI, low intensity; HI, high intensity; HIIE, high intensity interval exercise; IBFR30, intermittent blood flow restriction; CBFR, continuous blood flow restriction*.

### Time to Exhaustion

Four weeks of training with BFR increased the tolerance to severe exercise, but an increase was not observed in the exercise without BFR group in a study where Three men and ten women, physically active, were divided into two groups: interval training with BFR (*n* = 9) and interval training without BFR (*n* = 4). The training for both groups consisted of two sets of five to eight repetitions for 2 min at 30% of maximum power (P_má*x*_) with 1-min rest interval between the repetitions for 4 weeks Corvino et al. (2014) (Table 1).

### Maximum Power and Onset Blood Lactate Accumulation

In a study by de Oliveira et al. (2015), 37 physically active subjects, including 22 men and 15 women (23.8 ± 4 years), performed cycling training on a cycle ergometer three times a week for 4 weeks. The subjects were divided into four groups: (1) low-intensity interval training with BFR (LIIT + BFR; *n* = 10) performed at 30% of P_max_; (2) low-intensity interval training (LIIT; *n* = 7) performed at 30% of P_max_, (3) high-intensity interval training (HIIT; *n* = 10) performed at 110% of P_max_ and (4) high-intensity interval training combined with BFR (HIIT + BFR; *n* = 10) performed 50% as HIIT and 50% as BFR. Both groups performed two sets of 5–8 repetitions with 5-min intervals. The LIIT + BFR group improved the onset blood lactate accumulation by 16% and maximal power by 15% without a significant difference between the HIIT group and HIIT + BFR group (Table 1).

### Exercises, Sample Size, Gender, and Standards in the Studies

The 20 training protocols of AE + BFR that were employed to investigate the metabolic variables are highlighted: (a) Seven studies (35%) involved walking; (b) ten studies (50%) involved cycling; (c) two studies (10%) involved running and (d) one study (5%) involved running and walking. It was observed that 10 studies (50%) used treadmill, nine studies (45%) used cycle ergometer and one study (5%) use bicycle. In addition, eight studies (60%) involved continuous aerobic exercise protocols and 12 studies involved interval aerobic exercise protocols. Given that sample size and gender may be important aspects of a study, the sample sizes in those studies were small and ranged from nine to 37 subjects, with a predominance of males. Thirteen studies (65%) consisted of only men and the samples of seven studies (35%) involved both men and women. Regarding the device used for BFR, seventeen studies (85%) used cuffs, with sizes that ranging from 5 to 18 cm, and three studies (15%) used elastic bands with a size of 7.6–10 cm. Eleven studies were performed utilizing a fixed BFR pressure from 140 to 230 mmHg for all subjects, and six studies employed BFR pressure percentages that varied between 40 and 100% of arterial occlusion pressure. The studies that investigated the chronic effects of AE + BFR were conducted with 2–8 weeks of training in basketball players, young adults and the elderly.

## Effect of ae + Bfr on Neuromuscular Variables

Based on the studies that investigated the impact of AE + BFR on neuromuscular variables, three studies analyzed the acute effects (Ozaki et al., 2014, 2015; Kim et al., 2015) and 11 studies analyzed the chronic effects (Abe et al., 2006, 2010a,b; Ozaki et al., 2011; Sakamaki et al., 2011; Keramidas et al., 2012; de Oliveira et al., 2015; Kim et al., 2016; Clarkson et al., 2017). Studies that investigated the acute effects of AE + BFR assessed intracellular signaling associated with hypertrophy and muscular adaptations (Ozaki et al., 2014; Kim et al., 2015), and studies that investigated the chronic effects of AE + BFR evaluated muscle power (Keramidas et al., 2012), muscle strength (Abe et al., 2006, 2010b; Ozaki et al., 2010; de Oliveira et al., 2015; Kim et al., 2016), muscle hypertrophy (Abe et al., 2006, 2010a,b; Ozaki et al., 2011, 2015; Sakamaki et al., 2011), and growth hormone (Abe et al., 2006; Ozaki et al., 2015) (Table 2).

**Table 2:** Synthesis of the results of studies that evaluate the acute effect of combined aerobic exercise and blood flow restriction on neuromuscular variables in young adult and elderly subjects.

**References**	**Subjects**	**Variable**	**Exercise protocol**			**Intensity/requency of training/uration of study**	**Exercise volume**		**Interval**	**PADE** **(mmHg)**	**BFR between sets**	**SW(cm)**	**Results**
			**EX**	**FE**	**VE**		**Sets or continuous**	**Repetitions or duration time**					
Abe et al., 2006	18 Men healthy adults	GH, MH, FM	WALK	TREADMILL	50 m/min	2× day/6× week/weeks	5 Sets	2	1 min	120–200	Yes	5.0	↑GH, MH and MS for the group with BFR compared to CG
Abe et al., 2010b	19 Elderly men and women	MS, MH, FC	WALK	TREADMILL	67 m/min	5× weeks/6 weeks	Continuous	20 min	–	160–200	Yes	-	↑ MS, MH, HR in the group with BFR compared to CON
Abe et al., 2010b	19 Young men	HM	CYC	CYCLE	–	40% VO_2max_ 3× week/8 weeks	Continuous	15 min with BFR 45 min CON	–	160–210	Yes	-	↑ TM in the group with BFR
Ozaki et al., 2010	23 Sedentary men and women	MH, FM	WALK	TREADMILL	–	40% HRmax /4× week/10 weeks	Continuous	20 min	–	120–200	Yes	5.0	↑ Strength and muscular hypertrophy in the group with BFR
Sakamaki et al., 2011	17 Healthy men	MH	WALK	TREADMILL	50 m/min	2× day/6× week/3 weeks	5 Sets	2 min	1 min	160–230	Yes	–	↑ in muscle area in the group with BFR
Keramidas et al., 2012	20 Young men and women	Muscular Adaptation	CYC	CYCLE	–	3× week/6 weeks	3 Sets	2 min/2 min/6 min	–	90	No	–	BFR training induces peripheral muscle adaptation
Ozaki et al., 2014	6 Healthy men	ERK1/2, MTOR, P38. MAPK, AkT, S6K1	WALK	TREADMILL	100 m/min	WALK + BFR WALK	Continuous	20 min	–	240	Yes	5.0	↑ Activation of intracellular signaling pathways
Kim et al., 2015	10 Men	Muscular activation	CYC	CYCLE	50–60 rpm	HI VO_2max_ LI + 40%BFR LI + 60%BFR CON	Continuous	HI−14 minLI + 40%BFR−28 minLI + 60%BFR- 30 min	–	50–210	Yes	5.0	↑ muscle strees with high intensity exercise
de Oliveira et al., 2015	37 young adults	MS	CYC	CYCLE	–	3× week/4 weeks	2 Sets of 5 to 8 reps	2 min	1 min/reps and 5 min/sets	140–200	No	18	↑ of MS in the group with BFR
Ozaki et al., 2015	7 Elderly men	GH and MH	WALK	TREADMILL	–	45% HRres with and without BFR	Continuous	20 min	–	50–200		5.0	No correlation between GH and MH.
Kim et al., 2016	31 Healthy men	MS, MH	CYC	CYCLE	–	3× week/6 weeks	Continuous	20 min	–	120–180	Yes	–	No difference between groups
Clarkson et al., 2017	19 Men and women	FC	WALK	CIRCUIT	4 km/h	4× week/6 weeks	Continuous	10 min	–	60% of BFR max	Yes	10.5	↑ in FC in the group with BFR

*BFR, blood flow restriction; EX, exercise; FE, form of execution; VE, velocity of execution; WALK, walking; CYC, cycling; RUN, running; CYCLE, cycle ergometer; min, minutes; s, seconds; CON, Control; PADE, pressure applied during exercise; SW, sleeve width; –, Not informed; ↑, significant increase; LI + BFR, low intensity combined with blood flow restriction; LI, low intensity; HI, High intensity. GH, Growth Hormone; MDF, Maximum Dynamic Force; HR, Heart Rate; mTOR, mammalian target of rapamycin; MAPK, Mitogen-activated protein kinase; GH, Growth Hormone; MH, Muscle hypertrophy; FC, Functional Capacity; MS, muscle strength; HI, High Intensity; REPS, Repetitions*.

## Acute Effects of AE + BFR

### Intracellular Signaling Associated With Hypertrophy

Ozaki et al. (2014) performed an analysis of muscle biopsies indicated that Erk 1/2 phosphorylation levels were significantly higher after exercise. However, only the leg that performed the exercise with BFR caused an increase in p38 phosphorylation. For the mTOR signaling pathway, no changes in the Akt, mTOR, or S6K1 phosphorylation levels were observed before or after walking. Thus, 3 h after exercise, the eEF2 phosphorylation level for the leg with BFR was significantly lower than the eEF2 phosphorylation level for the leg without BFR. Walking with BFR can activate the intracellular signaling pathways that are associated with muscle hypertrophy in young men.

### Growth Hormone

Two studies investigated the impact of AE + BFR on growth hormone concentrations. Abe et al. (2006) noted that after the training period there had been an increase in growth hormone was only observed in the group with BFR. On the other hand Ozaki et al. (2015), correlated hormonal responses and hypertrophy. They revealed that the change in growth hormone, insulin and noradrenaline levels did not significantly correlate with the hypertrophy caused by AE + BFR, which suggests that walking with BFR-induced elevation in these anabolic hormones, but may not impact muscle growth.

### Muscle Torque and Muscle Activation

No significant changes in torque were observed for the protocols with BFR 1 min post exercise, but there was an increase in muscle thickness of the quadriceps (~2 mm) in the high intensity and low intensity protocols with 40% of BFR higher than other protocols of the study (Kim et al., 2015). Muscle activation with high intensity cycling was significantly higher than muscle activation in the sessions combined with BFR (Kim et al., 2015).

## Chronic Effects of AE + BFR

### Muscle Strength, Hypertrophy, and Physical Performance Tests

In the study of Abe et al. (2006), after the training period, an ~4–7% increase in the thigh muscle volume and cross-sectional area and an 8–10% increase in the dynamic strength and maximum isometric force in the group that trained with BFR were observed. In another study (Abe et al., 2010a), the authors discovered that the cycling combined with the BFR protocol promoted 3.4–5.1% muscle hypertrophy and the isometric strength in knee extension tended to increase by 7.7%.

Walking with BFR improved the functional capacity, increased the isometric strength (11%) and isokinetic strength (7–16%) in knee extension and flexion, and caused an increase in the cross-sectional area of the thigh (5.8%) and the leg (5.1%) (Abe et al., 2010b). Improvements were also found in 23 sedentary subjects—five men and 18 women—that were randomized into two groups 1) continuous walking with BFR and 2) continuous walking without BFR. The training was performed for 20 min on the treadmill with 45% of HR_max_ 4 days a week for 10 weeks. The group that trained with BFR used an inflatable cuff positioned in the region near the thighs with a severe pressure of 200 mmHg for all subjects. Increases in the maximal knee joint strength (~15%) and thigh muscle cross-sectional area (3%) were observed (Ozaki et al., 2010).

Regarding muscle volume, a significant increase in the muscle volume of the proximal region of the thigh (3.8%) and leg (3.2%) was observed (Sakamaki et al., 2011). Also, improvements in muscle strength by 11% were observed after a training program with BFR (de Oliveira et al., 2015). As for trained college students (*n* = 31) that performed 20 min of cycling, three times a week for 6 weeks no significant difference was found in muscle strength between the groups with or without BFR (Kim et al., 2016).

Elderly men and women (*n* = 19) that performed walking at a speed of 4 km/h for 10 min around a circuit in a field with a distance of 667 meters showed significant 2.5- to 4.5-fold improvement for the 30 s sit to stand, 6-min walk test, timed up and go, and a modified Queen's College step test was observed for the BFR group when compared with the control group after the 6-week training program (Clarkson et al., 2017).

### Local Aerobic Muscle Adaptations

In the study by Keramidas et al. (2012), twenty untrained subjects were randomized into a control group that trained without BFR and an experimental group that trained with BFR. Both groups trained 3 days per week for 6 weeks with the same relative intensity, and each training session consisted of 2 min at 90% of VO2_max_ and 2 min of active interval training at 50% of VO2_max_. An incremental exercise test to exhaustion, a 6-min constant-power test at 80% of VO_2max_ (Sub80) were performed pre- and post-training. It is notable that the AE + BFR group exhibited more pronounced muscle deoxygenation than the CON group, measured with near-infrared muscle spectroscopy. The higher post-training muscle deoxygenation shows up the capacity of the exercising muscles to work with a higher supply of oxygen.

### Exercises, Sample Size, Gender, and Standards in the Studies

The 12 training protocols of AE + BFR that were employed to investigate the neuromuscular variables are highlighted: (a) seven studies (58.3%) involved walking and five studies (41.7%) involved cycling. It was observed that six studies (50%) used treadmill, five studies (41.7%) used cycle ergometer and one study (8.3%) use circuit exercise. Additionally, eight studies (66.7%) involved continuous aerobic exercise protocols and four studies (33.3%) involved interval aerobic exercise protocols. Given that the sample size and sex may be an important aspect in these studies, sample sizes were small and ranged from six to 31 subjects, with a predominance of males. Seven studies (58.3%) were conducted with only men, and five studies (41.7%) were conducted with men and women. Regarding the device used for BFR, all studies (100%) used the cuffs with sizes that ranging from 5 to 18 cm. The BFR pressure in the cuffs ranged from 50 to 230 mmHg during exercise sessions that were intermittently performed (without deflating the cuff between sets) or continuously performed, and only the study by Kim et al. (2015) determined the BFR pressure by estimating the thigh circumference. In the studies that investigated the chronic effects of AE + BFR, training was performed between 2 and 10 weeks by young and elderly adults.

#### Effect of AE + BFR on Hemodynamic Variables

Based on the assessment of the articles that investigated AE + BFR, nine studies evaluated the acute effect of this training method on hemodynamic variables (Abe et al., 2006, 2010a; Ozaki et al., 2010; Renzi et al., 2010; Loenneke et al., 2011; Kumagai et al., 2012; Karabulut and Garcia, 2015; Sugawara et al., 2015; Ferreira et al., 2016; Cirilo-Sousa et al., 2017; Barilli et al., 2018; Conceição et al., 2018; Silva et al., 2018, 2019; Thomas et al., 2018). Studies that investigated the acute effect of AE + BFR assessed cardiac output (Ozaki et al., 2010; Kumagai et al., 2012; Sugawara et al., 2015; Thomas et al., 2018), heart rate variables (Abe et al., 2006, 2010a; Ozaki et al., 2010; Renzi et al., 2010; Loenneke et al., 2011; Kumagai et al., 2012; Karabulut and Garcia, 2015; Sugawara et al., 2015; Ferreira et al., 2016; Cirilo-Sousa et al., 2017; Barilli et al., 2018; Silva et al., 2018, 2019; Thomas et al., 2018), stroke volume (Ozaki et al., 2010; Kumagai et al., 2012; Sugawara et al., 2015; Ferreira et al., 2016), blood pressure (Ozaki et al., 2010; Renzi et al., 2010; Kumagai et al., 2012; Karabulut and Garcia, 2015; Ferreira et al., 2016; Cirilo-Sousa et al., 2017; Barilli et al., 2018; Silva et al., 2018; Thomas et al., 2018), total peripheral resistance (Kumagai et al., 2012; Sugawara et al., 2015; Ferreira et al., 2016), and double product (Renzi et al., 2010; Ferreira et al., 2016) (Table 3).

**Table 3:** Synthesis of the results of studies that evaluate the acute effect of combined aerobic exercise and blood flow restriction on hemodynamic variables in athletes, young adults, and elderly individuals.

**References**	**Subjects**	**Variable**	**Exercise protocol**			**Intensity/frequency of training/study of duration**	**Exercise volume**		**Interval**	**PADE** **(mmHg)**	**BFR between sets**	**SW(cm)**	**Results**
			**EX**	**FE**	**VE**		**Series or continuous**	**Repetitions or duration time**					
Abe et al., 2006	18 Healthy youth	HR	WALK	TREADMILL	50 m/min		5 Sets	2 min	1 min	120–220	Yes	-	↑ HR during protocol with BFR
Abe et al., 2010a	19 Young men	HR	CYC	CYCLE	50 m/min	40% VO_2max_BFR 40% VO_2Max_	Continuous	15 min with BFR 45 min without BFR	–	160–210	–	5.0	↑ HR in protocol with BFR
Renzi et al., 2010	17 men and women adults	HR, CO, DP and SBP	WALK	TREADMILL	3.21 Km/h	–	5-Sets	2 min	1 min	160	Yes	–	↑ HR, BP and DP and ↓ SV during training with BFR
Ozaki et al., 2010	10 Men	HR, CO, SBP, DBP, MAP, SV	CYC	CYCLE	–	0–20–40–60% VO_2max_ with and without BFR	Continuous	4 min each stage	–	200	Yes	5.0	↑HR, CO, SBP, DBP, MAP and ↓ VEJ in training with BFR
Loenneke et al., 2011	10 Men and women	HR	WALK	TREADMILL	75 m/min	–	5 Sets	2 min	1 min	Elastic	Yes	7.6	↑HR in the exercise session with BFR
Kumagai et al., 2012	8 Men	HR, SBP, DBP, SV, BP, CO, TPR	CYC	BICYCLE	–	40% VO_2max_ with and without BFR	Continuous	30 min	-	200	Yes	5.0	↑ HR BFR and CON ↑ SBP and DBP in training with BFR until 10 min
Karabulut and Garcia, 2015	34 Obese men and women	HR, SBP, DBP	CYC	CYCLE	50 rpm	40% + BFR 60% + BFR without BFR 50 Watts	2 Sets	10 min	1 min	40%60%	Yes	5.0	↑ > HR and SBP in BFR protocols
Sugawara et al., 2015	15 Adult men and women	HR, SBP, DBP, SV, CO, TPR	WALK	TREADMILL	3.21 Km/h	WALK + BFR WALK	5 Sets	2 min	1 min	160	Yes	–	↑ > HR, SBP in protocol with BFR. SV and TPR is > for the control
Ferreira et al., 2016	21 Elderly men and women	HR, SBP, DBP, DP, SV, TPR, CO	WALK	TREADMILL	–	40% VO_2max_BFR 40% VO_2max_ 70% VO_2Max_	Continuous	20 min	–	50%	Yes	17.5	↑ HR and SBP in protocols BFR. DP in the protocol HI > LI and LI+BFR
Cirilo-Sousa et al., 2017	13 Men	HR, SBP, DBP	RUN	STATIONARY	–	50% HRMáx+BFR 50% HRMáx	5 Sets	2 min	1 min	80%	No	18	↑ HR, SBP and DBP in protocol with BFR
Conceição et al., 2018	12 Men	HR	CYC	CYCLE	60–70 rpm	40% VO_2Max_ + BFR 40% VO_2Max_	Continuous	30 min	–	80%	No	18	↑ HR in protocol AE+BFR
Thomas et al., 2018	18 Men	BP, HR, CO	CYC	CYCLE	–	HI LI LI + BFR	3 Sets	2 min	2 min active	80%	Yes	10	↑BP, HR, CO in protocol HI > LI + BFR and LI BP, HR, CO in protocol LI + BFR > LI
Barilli et al., 2018	18 Elderly women	HR, SBP, DBP	WALK	TREADMILL		HIAE LI LI+BFR	Continuous	–	–	130% SBP rest	Yes	9.5	HR, SBP, and DPB in protocol HIAE = LI + BFR
Silva et al., 2018	23 Men	HR, SBP, DBP	RUN WALK	TREADMILL		HIIE AE + BFR AE	6 sets continuous	90 s 18 min	90 s active-	50%	No	18	↓ SBP, DPB in protocol HIIE and AE + BFR
Silva et al., 2019	22 Men	HR	RUNWALK	TREADMILL		HIIE AE + BFR AE	6 set Continuous	90 s 18 min	90 s active	50%	No	18	↑HR in protocol HIIE But, AE + BFR > AE

*BFR, blood flow restriction; HR, heart rate; BP, blood pressure; SBP, systolic blood pressure; DBP, diastolic blood pressure; TPR, total peripheral resistance; CO, cardiac output; SV, stroke volume; DP, double product; EX, exercise; FE, form of exercise; VE, velocity of exercise; RPM, rotations per minute; WALK, walking; CYC, cycling; RUN, running; CYCLE, cycle ergometer; min, minutes; s, seconds; CON, Control; PADE, pressure applied during exercise; SW, sleeve width; –, Not informed; ↑, significant increase; LI + BFR, low intensity combined with blood flow restriction; LI, low intensity; HI, high intensity*.

## Acute Effects of AE + BFR

### Cardiac Output

The studies that investigated cardiac output (CO) did not observe significant differences between a BFR exercise session and a control session performed without BFR (Ozaki et al., 2010; Renzi et al., 2010; Kumagai et al., 2012; Ferreira et al., 2016). The study of Ozaki et al. (2010) did not identify a significant difference in AE of the same intensity with or without BFR but did reveal that the high intensity exercise yielded higher cardiac values than low intensity exercise and low intensity with BFR exercise.

### Heart Rate

The study of Ferreira et al. (2016), which was performed with elderly individuals, demonstrated that heart rate (HR) in exercise performed with HI is higher than the heart rate in exercise performed with LI and LI + BFR. Some studies reported that during exercise, the increase in HR in LI + BFR exceeded the increase in HR in LI exercise (Abe et al., 2006, 2010a; Renzi et al., 2010; Loenneke et al., 2011; Silva et al., 2018, 2019). The studies by Ozaki et al. (2010) and Karabulut and Garcia (2015) suggest a linear increase in HR with an increase in exercise intensity and suggest that the higher the BFR pressure, the higher the HR values during exercise.

### Stroke Volume

Some studies reported that stroke volume in the exercise session performed with BFR was lower than the stroke volume in the control exercise session without BFR (Renzi et al., 2010; Kumagai et al., 2012; Sugawara et al., 2015). Ferreira et al. (2016) investigated hemodynamic variables up to 30 min after exercise and discovered that stroke volume only decreased in the exercise with BFR session.

### Blood Pressure

Systolic blood pressure (SBP) and mean arterial pressure (MAP) increased immediately after the session AE + BFR (Ozaki et al., 2010; Renzi et al., 2010). However, it was observed that during the aerobic exercise with BFR, the SBP and MAP were significantly higher than in the control session without BFR (Renzi et al., 2010; Karabulut and Garcia, 2015; Sugawara et al., 2015; Thomas et al., 2018). Diastolic blood pressure (DBP) during in exercise with BFR was higher than the DBP in the control session without BFR (Sugawara et al., 2015). The study by Ozaki et al. (2010) did not reveal any significant differences in MAP when the exercise was performed at 20% of VO2_max_ between the exercise with BFR and the control session without BFR. However, when the exercise was performed at intensities of 40 and 60% of VO2_max_, the MAP values in the exercise combined with BFR was higher than the MAP values in the exercise without BFR. Barilli et al. (2018) showed that 30 min after aerobic exercise with and without blood flow restriction, SBP and DBP return to resting levels. In the studies by Cirilo-Sousa et al. (2017) and Silva et al. (2018) the hypotensive effect occurred after 50 min of aerobic exercise with BFR.

### Total Peripheral Resistance

Kumagai et al. (2012) revealed that the total peripheral resistance (TPR) in the BFR exercise session was significantly greater than the TPR in the session with the same intensity without BFR. In the study by Ferreira et al. (2016), which was performed with elderly individuals, significant differences among the HI, LI, and LI + BFR protocols were not observed during and after the session. In the study by Ozaki et al. (2010), when exercise was performed with 20% of VO2_max_, significant differences were not observed between exercise with BFR and the control session. However, starting at 40% of VO2_max_, TPR in the BFR session was higher than the TPR in the control session.

### Exercises, Sample Size, Gender, and Standards Used in the Studies

The 15 training protocols of AE + BFR that were employed to investigate the hemodynamic variables are highlighted: (a) eight studies (53.3%) involved walking; (b) six studies (40%) involved cycling, and one study (6.7%) involved running. It was observed that eight studies (53.3%) used treadmill, five studies (33.3%) used cycle ergometer, one study (6.73%) use bicycle and one study (6.7%) did not use any equipment. In addition, in eight studies (53.3%) continuous aerobic exercise protocols were performed and in seven studies (46.7%) interval forms of aerobic exercise protocols were performed. Given that sample size and sex may be important aspects, the sample size in these studies was small and ranged from eight to 34 subjects, with a predominance of males. Nine studies (60%) involved only men, five studies (33.3%) involved men and women and one study (6.7%) involved women. Regarding the device for BFR, fourteen studies (93.3%) used inflatable cuff sizes that range from 5 to 18 cm, and only one study (6.7%) was performed using 7.6-cm elastic bands. Six studies were performed utilizing a fixed BFR pressure from 120 to 220 mmHg for all subjects, and seven studies employed BFR pressure percentages that varied between 40 and 80% of arterial occlusion pressure, and one study used 130% of resting systolic blood pressure. The BFR pressure in the cuffs ranged from 50 to 230 mmHg during exercise sessions that were performed in intervals (without deflation between sets) or continuously performed.

## Discussion

### Acute Effects of AE With BFR on Metabolic Variables

The studies that investigated the impact of AE with BFR on metabolic responses reported increases in VO_2_ throughout the sessions and an increase in the magnitude of EPOC, which exceeds the VO_2_ and EPOC in sessions with the same intensity without BFR; these findings may be related to an increase in exercise intensity (Ozaki et al., 2010; Loenneke et al., 2011; Mendonca et al., 2015; Silva et al., 2019), cumulative oxygen deficit (Mendonca et al., 2015), level of BFR pressure applied in the exercise (Pfeiffer et al., 2019), and the increased activity of the leg muscles with restricted blood flow (Loenneke et al., 2011)

The increase in metabolic variables caused by BFR may be explained by the increase in ventilation, ratings of perceived exertion, heart rate, and lactate during exercise (Abe et al., 2006; Conceição et al., 2018; Silva et al., 2019), which influences the replenishment of oxygen reserves in the blood and muscles, ATP and creatine phosphate resynthesis, lactate removal, and body temperature after exercise and contributes to an increase in the magnitude of EPOC (Mendonca et al., 2015).

Another factor that seems to influence the increase in VO_2_ throughout exercise sessions is the BFR pressure level applied during exercise with BFR (Pfeiffer et al., 2019), as seen in the study by Karabulut and Garcia (2015). These authors evaluated obese subjects on the cycle ergometer and observed that the cycling session performed with 60% of arterial occlusion pressure increased the energy demand more than a session performed with 40% of arterial occlusion pressure and wich increased the energy demand more than a cycling session without BFR. Thus, the increase in the energy demand in sessions of AE with BFR may depend on the BFR pressures that are applied during exercise. This increased energy demand may help to explain other physiological changes, such as heart rate and ventilation during aerobic training sessions with BFR (Conceição et al., 2018; Silva et al., 2019).

AE with BFR may cause greater recruitment of the leg muscles (Loenneke et al., 2011; Kim et al., 2015), which can be explained by the reduction in oxygen and subsequent metabolic accumulation. Thus, this accumulation of metabolites is believed to increase muscle fiber recruitment via stimulation of group III and IV afferent pathways, which may cause alpha motorneuron inhibition and an increase in the recruitment of muscle fibers to maintain force and protect against conduction failures (Loenneke et al., 2011). However, the lactate accumulation in AE with BFR is not a universal finding since Loenneke et al. (2012) discovered that low-intensity interval walking performed with a combination of elastic wraps did not promote metabolite accumulation due to the different amounts of BFR applied to the participants, which are influenced by the structure of the body segment, such as thigh circumference and limb composition.

Regarding the acute effects of AE with BFR, the study of Mendonca et al. (2014) reinforces the idea that an increase in the net VO_2_ throughout the AE with BFR session may occur due to an increase in the recruitment of type II fibers. This hypothesis is supported by some studies of the low load resistance training literature utilizing BFR, which have reported increases EMG amplitude (Yasuda et al., 2006; Karabulut et al., 2010). Karabulut and Garcia (2015) determined that the increased dependence on carbohydrates during exercise with BFR may be explained by increased adrenaline and noradrenaline levels and a greater demand for type II muscle fibers.

Thus, recent studies suggest that walking with BFR increases the electromyographic activity of the vastus lateralis; these findings are compatible with an additional recruitment of type II muscle fibers (Mendonca et al., 2015). Considering that type II muscle fibers require more O_2_ than type I for the same amount of work (Mendonca et al., 2015), fiber type recruitment has an important role in increasing VO_2_ during AE with BFR. Although it has not been explored, increased activation of the gluteus maximus may also contribute to an increase in the net VO_2_ during exercise with BFR.

### Chronic Effect of AE With BFR on Metabolic Variables

Regarding the chronic effects of AE with BFR, the adaptive responses that are linked to prolonged severe exercise tolerance after cycling training with BFR seems to be attributed to the increase in physiological and metabolic stress induced by BFR (Corvino et al., 2014; Conceição et al., 2018). Thus, the determinants of the time of exhaustion also seem to be related to the exhaustion of finite anaerobic reserves and the accumulation of a variety of metabolic by products. Park et al. (2010) observed a 2.5% increase in the anaerobic capacity of the group that trained with AE combined with BFR; this improvement can be attributed to an increase in the glycogen content, as observed in other studies with BFR (Sundberg, 1994; Burgomaster et al., 2003).

In the same study, Park et al. (2010) discovered an increase in the cardiorespiratory capacity (9–10% VO2_max_) in basketball players within 2 weeks; these results are similar to previous studies that used 7–10 days of cycling at 60–70% of VO2_max_ (Spina et al., 1996; Mier et al., 1997). Thus, training with BFR induces similar improvements to exercise with greater intensity performed for the same period of time.

Abe et al. (2010a) observed an improvement in cardiorespiratory capacity after cycling training with BFR in young adults. This improvement may be attributed due to adaptations in muscle oxidation capacity (arterial and mixed venous blood oxygen (a-VO_2_) difference and systolic volume). In addition, increased muscle mass in the lower body, capillary density, and citrate synthesis activity may be associated with improvements in VO2_max_ in the BFR training group. However, some studies that performed AE combined with BFR did not observe improvements in the cardiorespiratory capacity in elderly individuals (Abe et al., 2010b) or young individuals (Kim et al., 2016).

In the study by Abe et al. (2010b), the exercise intensity and duration of the training program may have influenced the results. Although the elderly subjects walked 5 days a week, the speed used for the exercise was low (67 m/min) and insufficient for improving the cardiorespiratory capacity. The same outcome may have occurred in the study by Kim et al. (2016) due to the use of reserve HR for exercise prescription with BFR. Thus, reserve HR may not be appropriate due to the observed increase in HR with the application of BFR due to a decrease in venous return.

de Oliveira et al. (2015) observed that LIIT + BFR improved the onset of blood lactate accumulation by 16% and maximum power by 15% without a significant difference among the HIIT groups. Thus, it seems that this training method increases the respiratory capacity of the muscle and promotes angiogenesis due to ischemia and reperfusion because the cuffs were deflated during the interval periods during exercise and this intermittent hypoxia can cause additional effects in some responses. In the study by Taylor et al. (2016), BFR was employed after the sprints, and the running training with BFR post-exercise was efficient for increasing the cardiorespiratory capacity of trained youth. This finding may be attributed to increased skeletal muscle and capillary density via cell signaling induced by enhanced hypoxia given the increased expression of growth factor HIF-1α.

### Effect of AE With BFR on Neuromuscular Variables

#### Adaptation in the Muscle Strength

A review of the selected studies reveals that the increased muscle strength via AE + BFR is not a universal finding. In four studies, an increase of 7.7–15% in muscle strength was observed within a period of 3–10 weeks of training with BFR (Abe et al., 2006, 2010a; Ozaki et al., 2010; de Oliveira et al., 2015). Thus, an increase in muscle strength via this training method is explained by neural adaptations during training-induced hypoxia (Abe et al., 2010a) and also partly via factors that increase muscle hypertrophy such as myogenic factors during exercise (Abe et al., 2006; Keramidas et al., 2012), however, the physiological mechanisms that explain the increase of muscle strength through AE + BFR need to be better elucidated. In only one study, muscle strength was not increased after an aerobic training program with BFR (Kim et al., 2016), which may have been attributed to the influence of the volume, intensity, and duration of training being lower than those from other studies.

### Adaptations in the Muscle Hypertrophy

Regarding the chronic effects of AE with BFR in muscle hypertrophy, it is known that this training method promotes muscle hypertrophy, however, this finding is not always observed. One study, which involved cycling with BFR, did not identify an increase in hypertrophy (Kim et al., 2016) after the training program. This can be explained by the use of very low training intensity (30% of HR_max_), lower than those from other studies. Thus, it has been demonstrated that performing 20 min of cycling, 3 times a week for 6 weeks using very low intensity training (30% of HR_max_) combined with BFR is not enough stimulus to promote adaptations in muscle strength and hypertrophy.

In addition, five studies investigating EA + RFS reported an increase between 3 and 7% of muscle hypertrophy in the thigh and leg (Abe et al., 2006, 2010a,b; Ozaki et al., 2010; Sakamaki et al., 2011). However, the gains of muscle hypertrophy through AE + BFR are not the same as the ones achieved by resistance training with BFR (RT + BFR). Studies with resistance training + BFR reported gains of 5–12.3% of muscle hypertrophy in the thigh (Takarada and Ishii, 2002; Abe et al., 2005; Kubo et al., 2006; Laurentino et al., 2008). Although gains in AE + BFR are lower than resistance training with BFR, the AE + BFR training method is still effective and can be used as a tool to maximize muscle strength and hypertrophy in young and old individuals.

In the study by Abe et al. (2010b), in which walking with a weekly frequency of 5 days was performed for 6 weeks, an increase in hypertrophy for thigh (5,8%) and lower leg (5,1%) muscles was observed in a group of elderly subjects who trained with BFR. Ozaki et al. (2010) found an increase in thigh hypertrophy (3%) in young individuals after 10 weeks an AE + BFR training program. In the study by Abe et al. (2010a), a cycling training program with BFR (40% of VO2max) with a duration of 8 weeks and a weekly frequency of three times a week promoted increase (3.4–5.1%) in hypertrophy in 19 young men. In the studies by Abe et al. (2006) and Sakamaki et al. (2011) the subjects underwent a low-intensity walk combined with BFR twice a day at a weekly frequency of 6 days for 3 weeks. It was observed that in the study by Abe et al. (2006) there was an increase of 4–7% in hypertrophy and in the study by Sakamaki et al. (2011) there was an increase of 3.2–3.8% in hypertrophy.

This increase in the hypertrophy may be attributed to increased levels phosphorylation of Erk1/2 and p38 (Ozaki et al., 2014), and increase in protein synthesis in the vastus lateralis muscle and the Akt/mTOR signaling pathway in young and elderly men, i.e., anabolic responses may significantly contribute to muscle hypertrophy induced by training with BFR. Still, in relation to the physiological mechanisms that explain the increase of hypertrophy through AE + BFR, it is worth noting that up until now the increase of hypertrophy cannot be explained by the elevation of growth hormone levels. In the study by Ozaki et al. (2015), there was no correlation between growth hormone and muscle hypertrophy, this reveals that elevations in growth hormone levels may not have a prominent role in muscle hypertrophy induced by walking with BFR.

Another important point that does not appear to influence the gains of hypertrophy through AE + BFR is muscle fatigue. Thus, regarding low intensity aerobic exercise in combination with BFR, Ogawa et al. (2012) observed no decrease in torque following slow or fast walking, suggesting that muscle fatigue may not be a prominent mechanism behind the skeletal muscle hypertrophy observed with low intensity aerobic exercise.

The results by Ogawa et al. (2012) corroborate the findings of Kim et al. (2015) that did not find significant changes in post-exercise torque, i.e., regardless of the intensity or pressure applied, the muscle was able to recover to a certain extent. Nevertheless, this is in contrast to the large decreases observed after exercise with resistance training (Loenneke et al., 2013). This large acute decrease immediately after resistance exercise appears to be evidence of fatigue and is thought to provide at least part of the mechanistic rationale for BFR inducing skeletal muscle hypertrophy when it is combined with resistance exercise (Loenneke et al., 2010).

### Adaptations in the Physical Performance Tests

Regarding the chronic effects of AE + BFR in the physical performance tests, it is observed that this training method promotes improvement of physical performance tests in elderly. In the study by Abe et al. (2010b) occurred improvement of physical performance and an increase in the isometric and isokinetic strength of the lower limbs in elderly after 6 weeks of AE + BFR. Furthermore, the study by Clarkson et al. (2017) found a positive correlation between increased knee extension strength and physical performance tests (for the 30 s sit to stand, 6-min walk test, timed up and go, and a modified Queen's College step test) following an aerobic training program with BFR. Therefore, it seems that a possible explanation for this adaptation to the AE + BFR would be the increase of muscle strength in elderly.

### Effect of AE With BFR on Hemodynamic Variables

Based on the evaluation of the studies that investigated the impact of aerobic exercise with BFR, the increase in HR, double product (DP) and SBP, DBP, and MAP during AE with BFR is greater than the increase observed in a session with the same intensity without BFR (Abe et al., 2006, 2010a; Ozaki et al., 2011; Kumagai et al., 2012; Loenneke et al., 2012; Silva et al., 2019). This finding can be partly explained by the level of BFR pressure during exercise, such that the pressure exerted by the cuff increases blood accumulation in the limbs and affects the strock volume due to a decreased venous return and elevation of noradrenaline concentration, which increases the peripheral vascular resistance (Ozaki et al., 2010).

Karabulut and Garcia (2015) observed a significant increase in SBP during cycling with BFR compared with a session of the same intensity without BFR. The phenomenon was attributed to an increase in cardiac contractility, which was caused by sympathetic stimulation or an increase in peripheral resistance when using BFR during exercise. Ferreira et al. (2016), Cirilo-Sousa et al. (2017), and Silva et al. (2019) indicated that following the post-exercise recovery period, the reduction in HR, DP and BP in the walking protocol with BFR was greater than the reduction in HR, DP, and BP in a session with the same intensity without BFR, with the autonomic cardiac modulation followed the same pattern. This finding was probably attributed to the increase in parasympathetic reactivation that was observed after the training session with BFR (Ferreira et al., 2016). These results have important clinical implications because the DP is well-correlated with the oxygen consumption in the myocardium, which demonstrates lower cardiovascular stress after the session with BFR.

### Standardization in the Interventions

The results of this review indicate that AE + BFR is an effective method to improve cardiorespiratory capacity, muscle strength, and hypertrophy of young and old individuals, despite the use relatively low training intensities. Most studies were conducted with healthy young men and the samples varied from 6 to 37 subjects in the investigations. In addition, it is difficult to compare the studies because most of them used training sessions with different intensities and duration. Furthermore, the studies also used a variety of methods to apply BFR in exercise protocols. Some studies used elastic bands, other studies used fixed restriction pressures for all subjects, and present large differences in occlusion cuff width.

To illustrate this point, some studies submitted subjects to walk or cycling using elastic bands, also known as practical BFR (Loenneke et al., 2011, 2012). Although the use of elastic bands allows for the practical application of BFR outside the laboratory setting, it is impossible to determine the pressure that is being applied to each subject included in the study. Other studies have used the approach of applying a fixed (constant) restrictive pressure to all subjects (Abe et al., 2010b; Renzi et al., 2010; Mendonca et al., 2014). Applying the same amount of pressure to all subjects without considering other factors such as limb size and composition may limit the interpretation of outcomes from these studies because a fixed pressure may elicit different degrees of occlusion across different individuals. A better approach was first proposed by Laurentino et al. (2008) who determined the BFR pressure individually for each subject based off the total auscultatory occlusion pressure for the limb as measured at rest through Doppler ultrasound.

### Suggestions for Future Research

We suggest that future studies investigate the effects of AE + BFR, acute changes and effects of a longer training period, in other variables not necessarily related to the muscle, such as blood pressure, sympathetic activation and vascular function, especially in clinical populations (i.e., people with hypertension, COPD, diabetes). That will provide more information about the impact of BFR in other systems of the body and would add knowledge about the safety of this type of exercise.

## Conclusions

Evidence reveals that AE combined with BFR promotes positive acute and chronic changes in neuromuscular and metabolic variables, a greater elevation in hemodynamic variables and a higher energy demand during and after exercise when compared to sessions of AE with the same intensity without BFR. The BFR was reported as well-tolerated in the populations targeted by the studies used in this review (i.e., athletes, healthy young individuals, obese and elderly individuals) since the increases hemodynamic variables during AE + BFR were smaller than high intensity training. However, these results may not be same in populations other that the ones covered by this review. In addition, the characteristics of the exercises used (i.e., form of execution, exercise intensity, determination of BFR pressure, and width of cuff or elastic band) were not standard among the studies, which may have caused the different acute and chronic responses to the AE with BFRE.

## Author Contributions

JS, GN, and EP participated in protocol design and data extraction. PP and AR in participated data analyses. MC-S, MB, SP, and GB participated in the preparation and review of the manuscript. All authors have read and approved the final manuscript.

### Conflict of Interest

The authors declare that the research was conducted in the absence of any commercial or financial relationships that could be construed as a potential conflict of interest.
